# Television as a Career Motivator and Education Tool: A Final-Year Nursing Student Cohort Study

**DOI:** 10.3390/ejihpe10010026

**Published:** 2019-12-24

**Authors:** Daniel Terry, Blake Peck

**Affiliations:** School of Nursing and Healthcare Professions, Federation University Australia, 3350 Ballarat, Australia; b.peck@federation.edu.au

**Keywords:** career choice, nurse, student, television, teaching, learning

## Abstract

Fictional medical programs are often used for more than just their recreational enjoyment; they can also influence career decision making. Very little research has examined the pedagogical value of fictional medical programs in terms of their motivational value in the choice of a nursing career. As such, the aim of this study was to examine what motivated nursing students to choose nursing careers, if fictional medical programs were motivators, and if they are used by students as a learning tool. The cross-sectional study collected data using a questionnaire and occurred between April and June 2018. The findings were generated from students’ short answers and extended responses within the questionnaire. Data were analysed using descriptive and inferential statistics, while qualitative data were analysed thematically. A total of 291 students participated (82.6% response rate), with motivations for entering nursing being similar to other international studies; however, as motivators, fictional medical television programs were rated higher than job security. Overall, students engage with medical television programs along a television–learning continuum, ranging from limited watching time, recognising inaccuracies, understanding dialogue, through to using fictional medical television programs as tools for learning. However, this is dependent on time, interest, current level of understanding, and a program’s perceived value.

## 1. Introduction

The motivation to enter the nursing profession has been well researched and is encompassed by the desire to help people, the altruism of the individual, financial drivers of the career outcome, and other factors which influence the individual to follow the career pathway [1]. The perception of what nurses are—caring professionals who have desire to make a difference—is one of the most reported motives for entering the profession [2,3]. It is this perception of the angelic, nurturing, and compassionate roles—now synonymous with the idealised version of the nurse—that is often at odds with the reality upon entering the profession, and it is this idealism that has been shown to exert a powerful influence on students completing their studies and the neophytes continuing in profession [2,4,5].

Despite what we already know about the influence of this idealised model of the nurse as a motivator for entering the profession, the most recent research has shown that people from other healthcare professions—including nursing—are influenced by fictional medical programs [6,7,8]. Popular media, specifically television, can enhance or impede how the public perceives the ways in which health professionals, including nurses, should act and behave. It is these same narrow stereotypes that can influence the decisions to enter the profession and which can be—and often are—reinforced among those studying the profession [7,9].

It is through these images that are used in popular media, specifically television, where medical and nursing students, as well as those contemplating enrolling in a respective degree program, begin to develop their professional identities, belief systems, and expectations as health professionals [7,9,10]. As such, students in these studies have been shown to be great consumers of the fictional medical programs not only for the recreational enjoyment, but also due the perceived relevance to their discipline of study [9,10,11]. A closer examination of the use of fictional media in health education reveals details of its use. Some studies—Shevell, Thomas, and Fuks [12] and Jubas and Knutson [13], for example—used short clips from a variety of common television media as teaching tools for demonstrating important clinical competencies, as well as modelling professional behaviours for medical interns. Another study by Williams and Ozakinci [14] examined the viewing habits of students and their recall of ethical dilemmas that arose as a vehicle for learning. In fact, these authors [14] suggest that fictional medical television media represent an important influence on medical students (future clinicians) by way of what has been termed the “hidden curriculum” (p. 55) of ethical development.

Nursing is a practice-based discipline, where clinical education forms an essential component of any nursing curriculum. At the centre of clinical experiences is the progression towards the development of self-efficacy, which is an individual’s sense of his or her capacity to execute behaviours for performing a given task or to enact particular types of behaviours [15]. One’s level of self-efficacy is an important predictor of performance in both the present and into the future; according to Bandura, television media—albeit fictional—might offer students an opportunity to enhance their self-efficacy [16]. His ideas of vicarious or visualised experiences of others’ accomplishments have been shown to have an impact on self-efficacy, as evidenced recently in a study on expectant mothers who experience changes in their self-efficacy when watching portrayals of birthing on television [17]. Seeing others perform activities or successfully achieving goals—including when portrayed on television—provides opportunities for social comparison, engendering expectations among observers that “If they can do it, so can I”. Seeing others’ successful performances and outcomes increases the observer’s sense of self-efficacy. While this modeling is often less powerful for developing self-efficacy, it is dependent on the context or situation in which it is used [15]. For example, a nursing student may gain greater self-efficacy from vicarious experiences that are observed in the classroom, in clinical settings, or even on the television prior to their own actual nursing performance [15,18].

Despite these examples of studies with positive outcomes, the pedagogical value of fictional medical programs is often mixed, with dichotomous language being used to describe them as both helpful and harmful [6,9,11]. They have been described as helpful in teaching key aspects of what to do and what not to do, but also as harmful, in that fictional medical programs may reinforce medical paternalism, the fallacy that poor ethical behaviour is acceptable, and that life-or-death situations are a dichotomy rather than a multiplicity of choices [11,19]. Very little research has examined the influence of fictional medical television programs on the choice of a nursing career or as a source of learning [6,7]. As such, the aim of this study was to examine what motivated final-year nursing students to choose a nursing career, if fictional medical television programs were a motivator, and if so, how their watching habits and television’s influence on learning may have changed over time.

## 2. Materials and Methods

A cross-sectional design was used to examine what motivated final-year nursing students to become Registered Nurses and if fictional medical programs were a motivator.

### 2.1. Setting

The study was conducted through an Australian university which has campuses in rural, regional, and peri-urban centres, which provides for a wide range of views regarding the motivations behind entering the profession.

### 2.2. Sample

All final-year nursing students (n = 352) studying for a three-year bachelor’s degree at an Australian university were invited to complete a questionnaire that examined their motivations for entering the nursing program to become Registered Nurses.

### 2.3. Data Collection Tool

Data were collected using a questionnaire that included demographic questions concerning age, rural background, and if they were first in their family to attend a university. In addition, the questionnaire included questions pertaining to when they first thought about becoming a nurse, when they took action to become a nurse, and factors that may have impacted their decision-making. Key questions were also asked if fictional medical programs on television were motivational factors in wanting to become a nurse. The questionnaire took between 5–10 min to complete.

### 2.4. Data Collection

Data collection occurred from April to June 2018, where students were provided with a copy of the questionnaire and were informed that by completing the voluntary questionnaire, they were consenting to participate as subjects in the research.

### 2.5. Ethical Considerations

Ethical approval was provided by the University’s Human Research Ethics Committee (A18-017).

### 2.6. Data Analysis

Quantitative data were cleaned, checked, and analysed using the Statistical Package for the Social Sciences (SPSS, Version 24.0) [20]. Descriptive statistic and Chi-square tests were used to explore if the age groups and where students grew up had correlations with student motivation. Significance was determined at two-tailed *p* ≤ 0.05.

Qualitative data were generated from the extended responses within the questionnaire, and a deductive thematic approach was selected to be used in the study to allow a simple method of systematically identifying recurring themes. A number of quotations are included to illustrate and support the findings that emerged from the textual responses. The important points and issues emerging from the qualitative data were identified and are discussed in detail.

## 3. Results

Among the third-year nursing students invited to participate, 291 responded, yielding a response rate of 82.6%. Table 1 outlines the demographic characteristics of the participants and highlights that over three quarters (77%) of the participants were between 20 and 29 years of age, with over a third (38.8%) of all participants having grown up in a small town with less than 20,000 people. More than half (57.7%) were the first in their families to attend university, and over a third (37.7%) had thought about or considered being nurses between 15 and 18 years of age, while over a third (39.9%) had committed to becoming nurses as adults.

In addition to when students first thought about and committed to becoming nurses, the motivations that lead them to make these decisions were varied. As outlined in Table 2, most students (70.8%) indicated that they wanted to help people, followed by wanting to make a difference (60.1%); however, the decision was also based on a health event that the participant or a family member had experienced, which included childbirth (37.1%). Others indicated that it was a family member who was a nurse which was the impetus to choose the profession as a career (31.3%), while others were motivated by the profession’s flexibility (28.9%), job security (26.8%), and the travel or adventure (26.8%) that the profession was perceived to allow. An interesting finding was that fictional medical programs on television were motivational in deciding to take up nursing as a profession in just under a third of all students (28.9%).

It was noted that there was no significance associated between fictional medical programs as a motivation and when the students first thought about becoming nurses (χ^2^ = 5.095, df = 3, *p* = 0.165), or the age at which they had committed to becoming nurses (χ^2^ = 5.215, df = 3, *p* = 0.157). The decision to enter nursing being based on fictional medical programs was not associated with the student’s age of decision about nursing itself, or what they had done to commence their studies to enter the profession. Furthermore, there was no significant association between fictional medical programs being a motivation and where the student grew up (χ^2^ = 5.674, df = 3, *p* = 0.225). From the data, it was shown that there was no association between fictional medical programs being a motivation for entering nursing as a profession and any other demographic factor.

Beyond the motivation of fictional medical programs, it was indicated that a third (33.9%) of the students watched fictional medical programs ‘often’ before commencing their Bachelor’s degree, while there were less than a quarter (23.4%) who watched ‘rarely’ or ‘never’. As outlined in Table 3, among students in their third year of higher education, it was revealed that under a quarter (33.9%) were watched fictional medical programs, while under a third (31.1%) watched less. However, there was a significant association between watching fictional medical programs prior to commencing higher education and the frequency of watching fictional medical programs as a nursing student (χ^2^ = 24.997, df = 3, *p* = 0.015). Those who watched fictional medical programs rarely or never prior to commencing higher education watched more as students (24.1%), while those who had watched more prior to higher education watched less as students (33.0%).

Students were asked to indicate which medical programs related to nursing, medicine, and health they watch or have watched in the past. It was indicated that *Grey’s Anatomy* was the most popular medical television show watched (49.1%), followed by *Offspring*, an Australian television medical drama (34.7%), and *The Good Doctor* (32.3%). This was followed closely by a number of other popular fictional medical programs, as outlined in Table 4. Interestingly, *24 h in A&E*, a British documentary program, was the only reality/real-life television show that was predominantly featured (11.3%) among student responses. There were ‘other’ reality/real-life television shows that were mentioned, including *one born every minute* (UK), *Embarrassing Bodies* (UK), *Botched* (US), and *RPA—Royal Prince Alfred* (Australia); however, these made up very few responses in total (9.6%).

**Figure 1:**
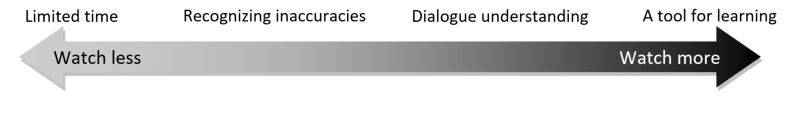
The television–learning continuum among nursing students.

In addition to regularity and types of medical programs that were watched, students were also asked to indicate why they were watching more, less, or the same number of medical programs prior to commencing their higher education nursing program. Within this question, participants discussed a number of factors, including the reasons behind why they did or did not watch medical television. Overall, there were four key themes that emerged from the responses; these are discussed below. Each theme is suggested to run along an education continuum, from limited time, dialogue understanding, recognising inaccuracies, to using medical programs explicitly as a tool for learning, as outlined in Figure 1.

### 3.1. Limited Time

In many cases, students indicated that the change in their medical program watching behaviours was due to limited time, much of which was centred on using what time they had on their studies or other essential or recreational pursuits such as employment, family commitments, and social activities. A discourse occurred among students concerning the ‘balancing’ of life and study, and for some students, television was not within the mix of activities considered to be vital. In this sense, students did not see television as an element of study or essential to the complexity and competing interests in their ‘life’; therefore, it is either removed or never included. However, some students would only add medical television to their ‘balancing act’ when other rival, yet important, activities were diminished or had contracted.

“I do not have enough time at the moment due to studies, family, and work, but I always do when I get the chance.”

(Student #270, Age 35)

“Because work commitments and study mean I am unable to, which doesn’t bother me.”

(Student #257, Age 23)

If time did allow, recreational television would often take precedence over medical television as a means of escaping, as demonstrated by the following comments.

“No time and also need to be able to ‘switch off’ from health care… so I watch less.”

(Student #146, Age 43)

### 3.2. Recognising Inaccuracies

Beyond limited time or the recreational escape from healthcare that television provided some students, other students indicated that they enjoyed watching medical programs; while many still watched the same amount, others had either decreased or increased their time watching. The reasons behind their decisions were varied and were often dependent on what they wanted to achieve from or avoid about the activity. For example, there were students who found that they enjoyed watching medical programs, but noted many were fictitious and unrealistic; others watched to actively pinpoint or highlight the inaccuracies that were occurring, as outlined below.

“I just enjoy that genre as they are generally dramas, and I am interested in the medical side, even if it is dramatized.”

(Student #290, Age 20)

“I have always been interested in that area and find them interesting, but know they are not accurate.”

(Student #012, Age 22)

“I watch more so I can see how many mistakes they make.”

(Student #45, Age 24)

“I find them interesting. It’s also fun to pick out the mistakes or the unrealistic situations.”

(Student #214, Age 21)

“Very fictional and frustrating because it’s not really what happens.”

(Student #228, Age 22)

In this sense, students enjoyed the drama of the medicalised program; others who were further along the television–learning continuum were slightly more analytical, investigative, and critical of the various inaccuracies that were occurring within each episode of the various programs. As such, students moved from disdain of the programs for their inaccuracies—leading to watching less—to enjoying the dramatized ‘stories’ couched within the medical programs, right through to the enjoyment of pulling programs apart and watching them even more.

### 3.3. Dialogue Understanding

Students indicated that an impetus for watching more medical programs was due to the development of their language and understanding of medical terminology. That is, as their language skills improved, their comprehension of the dialogue intensified, leading to their interest and ‘buying into’ the medical programs had increased. This was evident in the following statements:

“I can relate more to the shows and medical terms. I actually understand what they are talking about now.”

(Student #132, Age 20)

“Now I know what’s going on… I also understand the language better.”

(Student #189, Age 52)

“I can relate to them a lot more now; I can understand the terminology.”

(Student #244, Age 20)

Much of the discourse concerning this finding suggested that students felt that ‘now’ that there was clearer understanding, they could really move beyond the plot, drama, and characters within the program to be more enveloped by or focused on the medical or healthcare events occurring and encapsulated within the drama of each program’s episodes. The language used emphasised that students had stepped out of the darkness and into the light. They found greater meaning and depth to the programs they were watching. This led some students to indicate that they had even started to move beyond understanding and finding meaning to problem-solving medical issues as they watched their selected programs.

“I can understand the terminology used and sometimes help solve the cases in my head.”

(Student #116, Age 20)

“I understand what is going on and like to diagnose what they will be doing for the patient.”

(Student #060, Age 56)

### 3.4. A Tool for Learning

In addition to developing a greater understanding of the dialogue used and attempting to solve the medical issues in the medical programs, students explicitly highlighted that they used fictional medical programs as an educational vehicle to learn, be inspired, and stay motivated. This was emphasised by the following student statements:

“[A] better understanding of health conditions makes it even more interesting—it enhances my learning.”

(Student #160, Age 28)

“I watch more medical shows now to gain the understanding and knowledge; you can also start to relate to procedures being performed and the communication being used.”

(Student #246, Age 21)

“Although it’s just a series, I can still learn from it. E.g., clinical environment, relationships among practitioners, clients, [and] some general info as well.”

(Student #108, Age 22)

The learning that was being undertaken was suggested to be more than understanding medical terminology and knowing how to perform certain skills, but encompassed many aspects fundamental to nursing, such as communication, inter- and intra-professional interactions, and navigating the clinical setting. In addition, two students stated that watching medical programs “was almost [like] looking in a mirror” (Student #256, Age 21) and that this would help with “things I may come across or experience in the future” (Student #258, Age 21). This suggests that medical programs are recognised and used as a conduit to gain clinical knowledge while encountering vicarious experiences to develop the understanding of what it means to be a nurse and how to prepare for future practice.

Although fictional medical programs are used as a source of learning, students recognised the various programs’ own limitations due to the sometimes fabricated or contrived situations. To overcome this, students indicated that they would look beyond or filter out the counterfeit elements of the programs while distilling the key elements that could be used in their own experiences and practice in healthcare. One student stated:

“Some scenes [are] quite similar to the real stuff that is going on in the real hospital setting. So basically, just try to get the key images and parts out of the shows.”

(Student #003, Age 21)

In addition, some students indicated that they changed their own watching behaviours when they transitioned from fictional to more factual programs. Nevertheless, irrespective of the vicarious learning opportunity that watching television offered, students recognised that there were limitations. It was suggested that the process of watching could only achieve so much for students’ learning, and the activity needed to be enhanced with the application of what they had learned with adequate clinical supports in place. This was specifically highlighted by one student who stated:

“Whilst some learning can be gained from watching a real TV show, [real] experiences seem more beneficial because I have access to people to support my practice.”

(Student #195, Age 52)

Overall, students either saw fictional medical programs as a distraction, abhorrently unrealistic, something they could now more fully appreciate, or a medium that could be used to enhance learning. All perspectives were mediated by where students found themselves along the television–learning continuum, which may be dependent on how they viewed fictional medical programs, where they were situated in their own learning, and their capacity to see medical programs as another tool in their suite of learning equipment preparing them for and cultivating their practice as Registered Nurses.

## 4. Discussion

The overall motivations for entering nursing as a profession were found to be similar to those of other studies internationally, where the impetus was wanting to help others, personal health experiences, job security, recommendation or influence of significant others, or even not being able to get into other study programs [1,3,21,22]. This study also indicated that fictional medical television programs did motivate students to enter nursing, as outlined previously [6,7,10,23,24]. However, the magnitude at which medical television programs were considered a motivation has not been previously highlighted. In this case, television was the sixth highest factor—above job security—in motivating students to enter the nursing program, but was unrelated to the age of when they decided that nursing was their chosen career.

It was indicated that watching habits among the cohort had changed, where those who watched less prior to commencing their nursing studies now watched more, and those who watched more prior to commencing their nursing studies now watched less. Regardless of the changes in watching habits, medical television programs remain prominent in student viewing habits, as previously highlighted [10]. *Grey’s Anatomy* was the most watched program, which was also found in the study conducted by Weaver, Salamonson, Koch, and Jackson [7]. It was indicated that female students have a greater propensity to watch *Grey’s Anatomy* than their male counterparts, as outlined by Weaver and Wilson [9]. Given that the cohort of students was mostly female, this was anticipated, as students studying a healthcare degree are more likely to engage with medical television programs where the central character is of the same gender [9,10].

In addition to the watching behaviours and types of programs watched, students provided insight into why they watched more or less and the central reasons behind why they were watching. Weaver, Salamonson, Koch, and Jackson [7] and Czarny, Faden, Nolan, Bodensiek, and Sugarman [10] argue that medical television program provides a medium in which images and messages are embedded in the popular culture with inaccurate representations of reality and inform elements of the informal curriculum among nursing undergraduates. However, in this study, it is shown that there is a diversity of motives for watching or not watching medical television programs and that it is not always a passive or inactive process, where students may or may not critically engage with content or see value in the programs [7,9]. This study highlights that student are active participants who engage with medical television programs along a continuum. This television–learning continuum ranges from limited time to watch, recognising inaccuracies, understanding dialogue, and using medical television programs as tools for learning. Along the continuum, a student’s level of engagement is mediated by time, interest, current level of understanding, and the value they place on the programs.

As a motivator, the popular culture and depiction of nursing, whether good or bad and regardless of their relative accuracy, may be the initial driver for becoming nurses or entering nursing programs [5,7]. It has been suggested that nursing iconoclasts portrayed through media and popular culture have been the demise of people wanting to enter nursing [25,26]. However, within this study, medical television programs were shown to be a vehicle through which students were inspired to enter the profession and were a source of motivation within their current studies. Furthermore, it has been argued that the portrayal of realism and authenticity is often compromised or overlooked, as the narrative plays a central role; these flaws can lead to students emulating or basing clinical or ethical decision making on such inaccuracies [9,19,27]. Again, what this study highlights is that students are not credulous and merely accepting of medical television program content within popular culture, but that they are quite perceptive and more discerning than they are perhaps given credit for [7].

Weaver, Salamonson, Koch, and Jackson [7] have suggested that television may have an educational role; this study further indicates that it does more than has been appreciated. For example, students suggested that learning did occur when using medical television programs by developing medical lexicons, intra- and inter-professional communication, navigating the clinical environment, and developing critical thinking related to the physiological processes and being able to apply these in practical situations [28]. Television narratives provide broad and deep understanding of the human experience and ethical conundrums from both the healthcare practitioner’s and the patient’s perspectives, which has been suggested to be invaluable in healthcare teaching and learning [29,30,31]. However, medical television programs may also have implicit flaws where practices, such as resuscitation on television, may embed erroneous and fatal practices that have been shown to occur in practice. As such, medical television must be critically discussed and engaged within higher education to ensure that best practices are secured, as highlighted by Weaver and Wilson [9].

Interestingly, students did not explicitly discuss the absence of nurses as role models within the medical television programs, but focused more on the clinical practices. This may be due to the cohort of participants being final-year nursing students who may be focused more on completing their degrees. Ward and Summers [19] argue that little learning may be achieved for nurses through medical television; however, this study demonstrates that the invisibility of the nurse in many medical television programs may have little impact on learning. However, it may be argued that what students are seeking to learn from the programs is situated within, and continues to perpetuate the discourse of medical dominance and that nurses play subservient ‘feminine’ roles in healthcare. In addition, the failure among students to recognise and reflect on the roles of nurses is symbolic of the students’ inability to see their roles as both important and vital within the medical dominance discourse [19,32].

Regardless of the inherent limitations that medical television programs have, they are what Hoffman, Hoffman, Wessel, Shensa, Woods, and Primack [6] (p. 215) suggest as “an untapped resource that can serve as teaching (and recruitment) tools for students and [health] professionals.” Through this resource, along with other motivators, almost a third of nursing students are influenced to take up nursing. Once they commence their studies, medical television programs remain an easily accessible resource for students’ learning, regardless of what their learning needs may be, the nurses’ visibility in a program, or how programs portray nurses [7].

### Limitations

Overall, although the response rate for the questionnaire was more than 80% of the student cohort, there may be some limitations, as the study was only conducted among third-year nursing students and therefore may not be generalisable to all nursing students. First- and second-year students may have differing views regarding their motivations for becoming nurses, and they may have differing perceptions or practices concerning the use of television in their education. Furthermore, the study was conducted at one university which has campuses in regional and peri-urban locations with a relatively strong student cohort from regional and international settings, which may limit the ability to generalise the findings. In addition, students were asked about watching fictional medical television programs, which did not include other forms of media or social media they may use to inform learning, such as YouTube, Vimeo, and Netflix. Lastly, although the questionnaire provided open-ended questions, a greater in-depth qualitative understanding is required to grasp and further test the television–learning continuum, and would be valuable for future research.

## 5. Conclusions

The role of both fictional and reality medical drama on television can be viewed as helpful for nursing students to make sense of the world of patient care that they are about to enter. It is clear that the use of television to both orientate and consolidate learning in terms of clinical care and communication is a common trend amongst students in the 20–29 year age bracket, and for those who are the first in their families to study at an undergraduate level. This study, undertaken in a regional university setting in Australia, also identifies that students viewing more medical dramas on television report an increase in learning and sense-making about nursing as a career across a continuum. As students view more medical dramas on television while studying for their undergraduate degree, they report an increased ability to recognise inaccuracies in medical skills and actions in this study.

This study has identified four key themes, each running along a television–learning continuum, from limited time, dialogue understanding, recognising inaccuracies, to using medical programs as a tool for learning. Students who identified ‘limited time’ along this continuum did not see television as an integral part of their nursing study and tended to remove it from the balance in their lives, but added it when time allowed. Some students in this study indicated that they enjoyed watching medical programs and incorporated them into their lives. This study found that some students continued to enjoy watching medical programs and often recognised that inaccuracies were occurring in the dramatizations. The dialogical phase of the continuum would suggest that understanding and the extension of a student’s medical vocabulary enabled a deeper engagement with progressively more complex dramatized scenarios through their television viewing. In addition, students highlighted that they used and developed critical thinking and clinical reasoning in order to engage positively with the medical television programs as vehicles to learn, to be inspired, and to stay motivated.

While this study focused on final-year nursing students in an Australian regional university, it did not explore the impact that medical television viewing has on first- and second-year nursing students. The role of viewing medical dramas on television for current and prospective student nurses certainly warrants further investigation within these earlier year levels. It is clear that the role of medical television viewing in helping nursing students to understand, question, consolidate, and learn informally cannot be overlooked. Therefore, the implications of using medical television as both a motivator for potential students and a learning tool for current students need to be considered in both curriculum design and marketing initiatives for attracting nurses to the health workforce. As nurses are vital to health workforce initiatives across the globe, this study has certainly raised questions for further exploration.

## Figures and Tables

**Table 1:** Participant demographics.

Demographic Information	Frequency	Percentage (%)
Age (years) (n = 289)		
- 20–29 years	198	68.5%
- 30–39 years	51	17.6%
- 40–49 years	30	10.4%
- 50 years and over	10	3.5%
Where participant grew up (n = 281)		
- Inner City Metropolitan	18	6.4%
- Outer Suburb Metropolitan	40	14.2%
- Large Regional Centre	62	22.1%
- Small Town	109	38.8%
- On a Property or Farm	41	14.6%
- Other	11	3.9%
First in family to attend university (n = 291)	168	57.7%
Age when first thought about becoming a nurse (n = 284)		
- Under 15 years of age	26	9.2%
- 15–18 years of age	107	37.7%
- 19–25 years of age	85	29.9%
- Over 25 years of age	66	23.2%
When committed to be a nurse (n = 289)		
- Before Primary School	11	3.8%
- Primary school (Prep to grade 3)	18	6.2%
- Primary school (grade 4–6)	14	4.8%
- High school (grade 7–10)	49	16.8%
- High school (grade 11–12)	81	27.8%
- As an Adult	116	39.9%

**Table 2:** Student motivations for wanting to become a nurse.

Rank	Student Motivation Information	Frequency	Percentage (%)
1	Wanted to help people	206	70.8%
2	Wanted to make a difference	175	60.1%
3	A health event that occurred to me or family member	108	37.1%
4	A nurse(s) in the family	91	31.3%
5	The job offers flexibility	84	28.9%
6	Fictional medical programs on television	82	28.3%
7	Wanted job security	78	26.8%
8	Opportunity to travel or adventure	78	26.8%
9	Wanted good/better income	60	20.6%
10	Always wanted to be a nurse	60	20.6%
11	Work experience/volunteering in a health setting	51	17.5%
12	I was born for it	28	9.6%
13	My family told me	27	9.3%
14	Changed university courses	16	5.5%
15	I did not really know what to study at university	13	4.5%
16	Could not get into medicine	6	2.1%

**Table 3:** Frequency of television watching before and after commencing nursing degree.

Frequency of Television Watching	Frequency	Percentage (%)
How often watched television was watched before starting degree		
- Often	97	33.9%
- Sometimes	122	42.7%
- Rarely	56	19.6%
- Never	11	3.8%
How often television was watched after starting degree		
- Much higher	24	8.7%
- Higher	41	14.9%
- About the same	125	45.3%
- Lower	60	21.7%
- Much lower	26	9.4%

**Table 4:** Type of television programs watched.

Type of Programs Watched	Frequency	Percentage (%)
Grey’s anatomy	143	49.1%
Offspring †	101	34.7%
The Good Doctor	94	32.3%
Scrubs	92	31.6%
ER	91	31.3%
All Saints †	90	30.9%
House	87	29.9%
The Flying Doctors †	45	15.5%
Mash	42	14.4%
Private Practice	38	13.1%
24 h in A&E *	33	11.3%
Chicago Med	32	11.0%
Nip/Tuck	29	10.0%
Other real life television programs *	28	9.6%
A Country Practice †	25	8.6%
Nurse Jackie	20	6.9%
Doc Martin	17	5.8%
General Hospital	17	5.8%
Casualty	16	5.5%
Chicago Hope	9	3.1%
Doogie Howser	5	1.7%
St. Elsewhere	2	0.7%

* Reality/real-life television, † Australian television programs.
